# Simultaneous attenuation of hyperglycemic memory-induced retinal, pulmonary, and glomerular dysfunctions by proinsulin C-peptide in diabetes

**DOI:** 10.1186/s12916-023-02760-7

**Published:** 2023-02-13

**Authors:** Hye-Yoon Jeon, Chan-Hee Moon, Eun-Bin Kim, Nilofar Danishmalik Sayyed, Ah-Jun Lee, Kwon-Soo Ha

**Affiliations:** Department of Molecular and Cellular Biochemistry, Kangwon National University School of Medicine, Chuncheon, 24341 Kangwon-Do Korea

**Keywords:** Hyperglycemic memory, C-peptide, Diabetic complications, Vascular leakage, Neurodegeneration, Fibrosis

## Abstract

**Background:**

Hyperglycemic memory (HGM) is a pivotal phenomenon in the development of diabetic complications. Although coincident diabetic complications are reported, research on their development and treatment is limited. Thus, we investigated whether C-peptide can simultaneously inhibit HGM-induced retinal, pulmonary, and glomerular dysfunctions in diabetic mice supplemented with insulin.

**Methods:**

Insulin-treated diabetic mice were supplemented with human C-peptide by subcutaneous implantation of K9-C-peptide depots for 4 weeks, and reactive oxygen species (ROS) generation, transglutaminase (TGase) activity, and vascular leakage were examined in the retina, lung, and kidney.

**Results:**

We found hyperglycemia-induced persistent ROS generation and TGase activation after blood glucose normalization in the retina, lung, and kidney of insulin-supplemented diabetic mice. These pathological events were inhibited by systemic supplementation of human C-peptide via subcutaneous implantation of a thermosensitive biopolymer-conjugated C-peptide depot. ROS generation and TGase activation were in a vicious cycle after glucose normalization, and C-peptide suppressed the vicious cycle and subsequent endothelial permeability in human retinal endothelial cells. Moreover, C-peptide supplementation ameliorated HGM-induced retinal vascular leakage and neurodegeneration, pulmonary vascular leakage and fibrosis, and glomerular adherens junction disruption and vascular leakage.

**Conclusions:**

Overall, our findings demonstrate that C-peptide supplementation simultaneously attenuates vascular and neuronal dysfunctions in the retina, lung, and glomerulus of insulin-supplemented diabetic mice.

**Supplementary Information:**

The online version contains supplementary material available at 10.1186/s12916-023-02760-7.

## Background

Diabetes mellitus (DM) is a serious metabolic disorder characterized by chronic hyperglycemia with impaired metabolism of carbohydrate, fat, and protein resulting from a defect in insulin secretion or insulin action [1, 2]. Chronic hyperglycemia in diabetes is accompanied by high mortality and morbidity due to concomitant vascular complications, which are grouped into macrovascular and microvascular types [3]. Microvascular complications caused by damage to the small blood vessels include diabetic retinopathy (DR), diabetic kidney disease (DKD), and diabetic peripheral neuropathy (DPN) [2, 4]. DR, which affects about one-third of diabetic patients worldwide, is a leading cause of visual disability and blindness [5]. DKD is a serious and progressive complication that can lead to end-stage kidney disease [6]. Recently, it was suggested diabetes also induces pulmonary dysfunction as a microvascular complication [7, 8].

DM is a complex disease that develops in various complication states. The coincidence of diabetic microvascular complications such as DR, DPN, and DKD has been reported [9–11]. A number of epidemiological studies of type 1 and type 2 diabetes revealed high co-occurrence of DR and DKD [11–14]. Likewise, a clinical study showed the clustering of multiple diabetic microvascular complications such as DR, DKD, and DPN in type 1 diabetes [9]. The coincidence of DR and systemic vascular disorders, including DKD and cardiovascular disease, increases all-cause mortality compared to patients without either complication [13]. Therefore, therapeutic strategies to target multiple diabetic complications are needed to prevent additional complications after the occurrence of an initial diabetic complication and to reduce the risk of mortality in diabetes. Despite that, research on the development and treatment of coincident diabetic microvascular complications is limited.

The development and progression of microvascular complications depend on both the duration and the severity of hyperglycemia [1]. Treatment of diabetes has therefore focused on the management of blood glucose levels using oral hypoglycemic agents and insulin administration to hinder the onset and progression of microvascular complications [2, 15, 16]. Despite efforts to normalize hyperglycemia, progress in the treatment of diabetic complications has been hindered by the persistence of hyperglycemic stress after normoglycemia is restored, which is commonly referred to as hyperglycemic memory (HGM) [2, 17, 18]. HGM plays a pivotal role in the development and progression of diabetic vascular complications [3, 19, 20]. Clinical trials demonstrated HGM causes diabetic vascular complications to continue to progress after blood glucose normalization in patients with type 1 or type 2 diabetes [3, 21, 22].

The underlying mechanisms of HGM have been investigated to uncover the pathophysiology of diabetic vascular complications [2, 17, 19]. In the aorta of insulin-supplemented diabetic mice (HGM mice), protein kinase C βII continuously activates mitochondrial adaptor protein p66^Shc^ after the restoration of normoglycemia, resulting in the sustained generation of reactive oxygen species (ROS) and reduction of nitric oxide bioavailability [19, 23]. Sustained ROS generation upregulates the expression of NF-_k_B subunit p65 and Set7/9-mediated monomethylation of histone H3 at lysine 4, leading to the overexpression of inflammatory genes including vascular cell adhesion molecule-1 and monocyte chemoattractant protein 1 [19]. In the aorta of HGM mice, persistent ROS generation is in a vicious cycle with transglutaminase 2 (TGase2) and p66^Shc^, which is regulated by the transcription factor p53, and this vicious cycle plays a vital role in HGM-induced expression of endothelial inflammatory adhesion molecules and apoptosis [17, 23]. Therefore, intervention to arrest persistent ROS generation and TGase activation is important to inhibit the development and progression of HGM-induced vascular complications.

We hypothesized that systemic C-peptide supplementation can exert simultaneous inhibitory effects against HGM-induced retinal, pulmonary, and glomerular dysfunctions in HGM mice. Proinsulin C-peptide is reported to have beneficial effects against diabetic microvascular complications [1, 24]. We tested this hypothesis using human retinal endothelial cells (HRECs) and HGM mice. We found HGM-induced sustained ROS generation and TGase activation after blood glucose normalization, and these pathophysiological events were inhibited in three organs by subcutaneous implantation of K9-C-peptide, a human C-peptide conjugated to a thermosensitive elastin-like polypeptide (ELP). K9-C-peptide formed a depot, which provided the physiological range of human C-peptide into circulation for at least 2 weeks. Moreover, C-peptide supplementation ameliorated persistent microvascular and neuronal dysfunctions in the retina, lung, and glomerulus of HGM mice. Thus, our results suggest an approach for the simultaneous mitigation of multiple diabetic complications.

## Methods

### Generation of diabetic mice

Six-week-old male C57BL/6 mice were obtained from DBL (EumSeong, Korea). TGase2-null (*Tgm2*^*−/−*^) mice (C57/BL6), generated by disrupting exons 5 and 6 of the TGase2 gene [25], were kindly provided by Dr. Soo-Yul Kim (National Cancer Center, Goyang, Republic of Korea). Mice were maintained under pathogen-free conditions in a temperature-controlled room with a 12-h light/dark cycle. Diabetic mice were generated by single daily intraperitoneal injections of streptozotocin (50 mg/kg body weight; MilliporeSigma, Burlington, MA, USA) freshly prepared in 100 mM citrate buffer (pH 4.5) for 5 consecutive days, as previously described [26]. The control group mice were injected with an equal volume of the citrate buffer. Mice with fasting blood glucose concentrations ≥ 19 mmol/L, polyuria, and glucosuria were considered diabetic. Mouse blood glucose levels and body weights were monitored weekly.

### Treatment of mice with insulin, K8 polypeptide, and K9-C-peptide

Human recombinant insulin was systemically supplemented by subcutaneous implantation of osmotic pumps as previously described [17]. Briefly, 6 weeks after the first streptozotocin injection, diabetic mice were anesthetized with 3% isoflurane and implanted with an Alzet Mini-Osmotic Pump 2004 (Durect, Cupertino, CA, USA), which delivered human recombinant insulin (MilliporeSigma) at a rate of 58.4 pmol/min/kg. One day after implantation of the osmotic pumps, human C-peptide was systemically supplemented by subcutaneous implantation of K9-C-peptide depots as previously described [26]. Briefly, insulin-supplemented diabetic mice were subcutaneously injected into the nape of the neck two times for 4 weeks with 100 mg/kg K9-C-peptide or 83 mg/kg of K8 polypeptide, a negative control for the K9-C-peptide, in PBS. The K9-C-peptide and K8 polypeptide were prepared by inverse transition cycling as previously described [27]. Four weeks after the subcutaneous injections, sera and retinal, lung, and kidney tissues were obtained from the mice and analyzed. All animal experiments conformed to the *Guide for the Care and Use of Laboratory Animals* (National Institutes of Health; Bethesda, MD, USA) and were approved by the Institutional Animal Care and Use Ethics Committee of Kangwon National University (KW-201204–3).

### Measurement of insulin and C-peptide levels in mouse serum

Insulin and C-peptide levels in mouse serum were measured using a human insulin ELISA kit (MilliporeSigma, Burlington, MA, USA), a mouse C-peptide ELISA kit (Alpco, Salem, NH), and a human C-peptide ELISA kit (MilliporeSigma), respectively, according to the manufacturer’s instructions as previously described [26]. The blood levels (*n* = 6) of insulin and C-peptide were determined by measuring absorbance at 450 nm using a microplate spectrophotometer (Epoch; BioTek, Winooski, VT, USA).

### Measurement of ROS generation in mouse tissues

ROS levels in mouse tissues were determined using dihydroethidium (Thermo Fisher Scientific) as previously described [7]. Briefly, mouse retinas, lungs, and kidneys were dissected and embedded in optimal cutting temperature (OCT) compound (Sakura Finetek, Torrance, CA, USA). Unfixed cryosections (10 µm for the retinas, 40 µm for the lungs, and 20 µm for the kidneys) were prepared using a microtome-cryostat (Leica Biosystems, Wetzlar, Germany) and incubated with 5 µmol/L (retina) or 10 μmol/L (lungs and kidneys) dihydroethidium for 30 min at 37 °C. ROS generation was visualized by confocal microscopy and quantified by measuring the fluorescence intensities (*n* = 6).

### Measurement of in vivo TGase activity in mouse tissues

In vivo TGase activity in mouse tissues was determined as previously described [5, 7]. Briefly, unfixed cryosections (10 µm for the retinas, 40 µm for the lungs, and 20 µm for the kidneys) were incubated with 1 mmol/L 5-(biotinamido)pentylamine for 1 h at 37 °C. Retinal sections were then fixed with 3.7% formaldehyde for 30 min, and lung and kidney sections were fixed with cold acetone for 10 min. After fixation, the sections were permeabilized with 0.2% Triton X-100 in PBS and incubated with a blocking solution of 2% bovine serum albumin in 20 mmol/L Tris (pH 7.6). The samples were incubated with fluorescein isothiocyanate (FITC)-conjugated streptavidin (1:200, v/v) for 1 h and observed by confocal microscopy. TGase activities were quantified by measuring fluorescence intensities (*n* = 6).

### Measurement of vascular leakage in mouse tissues

Microvascular leakage in mouse retinas, lungs, and kidneys was measured as previously described [5, 7, 28]. Briefly, mice were deeply anesthetized, and 1.25 mg 500-kDa FITC-dextran (MilliporeSigma) was injected into the left ventricle and circulated for 5 min. Enucleated eyes were fixed with 4% paraformaldehyde (MilliporeSigma), and whole-mounted retinas were observed by confocal microscopy (K1-Fluo; Nanoscope Systems, Daejeon, Korea). Lung and kidney tissues were fixed with 4% paraformaldehyde and embedded in an OCT compound (Sakura Finetek). Cryosections (20 μm thick) were prepared using a microtome-cryostat and observed by confocal microscopy (K1-Fluo).

### Immunofluorescence staining

The expression of glial fibrillary acidic protein (GFAP) and glutamate aspartate transporter 1 (GLAST) in the retinal sections was visualized by immunofluorescence. After fixation and permeabilization, retinal sections were incubated with monoclonal antibodies against GFAP and GLAST (1:200; Cell Signaling, Danvers, MA, USA) overnight at 4 °C. The expression of the proteins was visualized by confocal microscopy and quantitatively analyzed by measuring the fluorescence intensities (*n* = 6).

### Western blot analysis

Western blot analysis was performed as previously described. Briefly, protein extracts from six retinas for each group were resolved by SDS-PAGE and transferred to polyvinylidene fluoride membranes. The membranes were incubated with monoclonal antibodies against GFAP and GLAST (1:2000; Cell Signaling) followed by incubation with horseradish peroxidase-conjugated secondary antibody. Protein bands were visualized using a ChemiDoc (Bio-Rad, Hercules, CA, USA).

### Measurement of neuro-apoptosis in mouse retinal sections

To assess neuro-apoptosis in mouse retinas, the retinas were stained using the APO-BrdU TUNEL assay kit (BD Bioscience, San Jose, CA, USA) as previously described [17]. Briefly, cryosections of retinas (10 µm thick) were fixed with 1% (w/v) paraformaldehyde and 70% (v/v) ethanol on ice. The fixed tissues were incubated with a DNA-labeling solution containing terminal deoxynucleotidyl transferase and 5-bromo-2-deoxyuridine in a reaction buffer for 1 h at 37 °C. The tissues were then incubated with an FITC-labeled 5-bromo-2-deoxyuridine antibody for 30 min and with 1 µg/mL DAPI (MilliporeSigma) for 10 min. The stained retinal sections were visualized using confocal microscopy (*n* = 6).

### Lung histopathological analysis

Lung fibrosis was assessed using hematoxylin and eosin (H&E) and Sirius red staining as previously described [8]. Briefly, lung tissues were fixed with 4% paraformaldehyde and embedded in paraffin. Lung Sects. (7 µm thick) were prepared using a microtome-cryostat (Leica Biosystems, Wetzlar, Germany), deparaffinized, and stained with H&E and Sirius red. Images were obtained by light microscopy (K1-Fluo). The fibrosis levels were analyzed by calculating the positive areas of Sirius red staining (*n* = 6). The positive area was defined by setting a threshold using the ImageJ software (NIH, Bethesda, MD, USA).

### Cell culture

HRECs were purchased from the Applied Cell Biology Research Institute (Cell Systems, Kirkland, WA) and grown on 2% gelatin-coated plates in M199 medium supplemented with 20% FBS, 3 ng/mL basic fibroblast growth factor, 100 U/mL penicillin, and 100 mg/µL streptomycin in a humidified 5% CO_2_ incubator. Cells were authenticated by short tandem repeat profiling, and subconfluent cells after 7–9 passages were used in experiments. For the experiments, endothelial cells were incubated for 12 h in a low-serum medium supplemented with 2% fetal bovine serum, 0.1 ng/mL basic fibroblast growth factor, and antibiotics. The cells were then treated with 5.5 mM d-glucose for 6 days (normal glucose), 30 mM glucose for 6 days (high glucose), or 30 mM glucose for 3 days followed by 5.5 mM glucose for 3 days (HGM).

### Measurement of ROS levels and in situ TGase activity in endothelial cells

Intracellular ROS levels were measured in HRECs using CellROX™ green reagent and 2′,7′-dichlorodihydrofluorescein diacetate (Thermo Fisher Scientific, Waltham, MA, USA) as previously described [17, 26]. Labeled cells on coverslips were mounted in a perfusion chamber and analyzed by confocal microscopy (K1-Fluo). ROS levels were determined at the single-cell level for 30 randomly selected cells in three microscopic fields per experiment (*n* = 4).

In situ TGase transamidating activity was measured by confocal microscopic assay as previously described [28]. Briefly, HRECs were treated with 1 mmol/L 5-(biotinamido)pentylamine for 1 h and fixed with 3.7% formaldehyde for 30 min. The cells were then permeabilized with 0.2% Triton X-100 in PBS for 30 min and stained with FITC-conjugated streptavidin (1:200; MilliporeSigma, Burlington, MA, USA) for 1 h. The fluorescence intensities of single stained cells were determined by confocal microscopy (K1-Fluo) for 30 randomly selected cells in three microscopic fields per experiment (*n* = 4).

### In vitro permeability assay

In vitro endothelial cell monolayer permeability was assessed as previously described [28]. Briefly, HRECs were grown to confluence on gelatin-coated inserts of Transwell Permeable Supports (0.4 µm, Costar, Corning, NY, USA) and incubated at 37 °C for 6 days in low-glucose, high-glucose, or HGM conditions. The treated cells were then probed with 1 mg/mL 40-kDa FITC-dextran for 60 min, and the amount of FITC-dextran in the lower chambers of the culture wells was measured by a fluorescence scanner (InnoScan 300, Innopsys, Carbonne, France) using well-type arrays (*n* = 4), fabricated by mounting PDMS gaskets onto clean glass slides as previously described [29].

### Statistical analysis

Data were analyzed using the OriginPro 2015 software (OriginLab, Northampton, MA, USA). Data are expressed as means ± standard deviation (SD) of six independent experiments. Statistical significance was determined using one-way ANOVA with Holm-Sidak’s multiple comparisons test. *P* values < 0.05 were considered statistically significant.

## Results

### Systemic C-peptide supplementation simultaneously inhibits persistent ROS generation and TGase activation in the retina, lung, and kidney of HGM mice

**Fig. 1:**
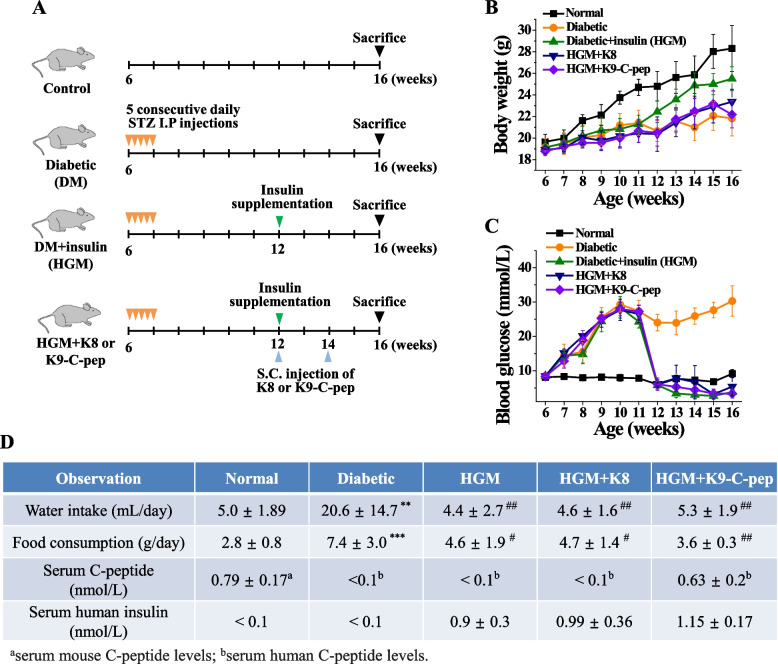
Biochemical parameters of normal, diabetic, and HGM mice and HGM mice supplemented with K9-C-peptide or K8 polypeptide. **A** Scheme of the generation of diabetic (DM) and insulin-supplemented diabetic (hyperglycemic memory (HGM)) mice and subcutaneous injection of HGM mice with K9-C-peptide (K9-C-pep) or K8 polypeptide (K8). **B**–**D** Six weeks after streptozotocin injection, diabetic mice were supplemented with recombinant human insulin using osmotic pumps for 4 weeks. One day after osmotic pumps were implanted, HGM mice were subcutaneously injected in the nape of the neck twice for a total of 4 weeks with K9-C-peptide (HGM + K9-C-pep) or K8 polypeptide (HGM + K8), a negative control for the K9-C-peptide. Body weight (**B**) and blood glucose levels (**C**) were monitored weekly (*n* = 6). **D** Ten weeks after streptozotocin injection, water intake, food consumption, and plasma levels of human insulin and C-peptide were determined (*n* = 6). **P < 0.01, ***P < 0.001 (vs. normal); ^#^*P* < 0.05, ^##^*P* < 0.01 (vs. diabetic). NS, non-significant

To investigate whether C-peptide can simultaneously inhibit retinal, pulmonary, and glomerular dysfunctions in HGM mice, diabetic mice were supplemented with human recombinant insulin and human C-peptide for 4 weeks (Fig. 1A). Compared with non-diabetic controls, diabetic mice showed decreased body weight, hyperglycemia, and elevated water and food consumption, all of which were normalized by insulin supplementation (Fig. 1B–D). Neither K9-C-peptide nor K8 polypeptide, a negative control for K9-C-peptide, had an effect on body weight, blood glucose level, or water and food consumption. Serum insulin levels were in the physiological range in HGM mice and HGM mice injected with K9-C-peptide or K8 polypeptide (Fig. 1D). Serum levels of C-peptide were 0.63 ± 0.2 nmol/L at 4 weeks after K9-C-peptide implantation, which is within the reference range, but were not detectable in the HGM mice or the HGM mice injected with K8 polypeptide (Fig. 1D).

**Fig. 2:**
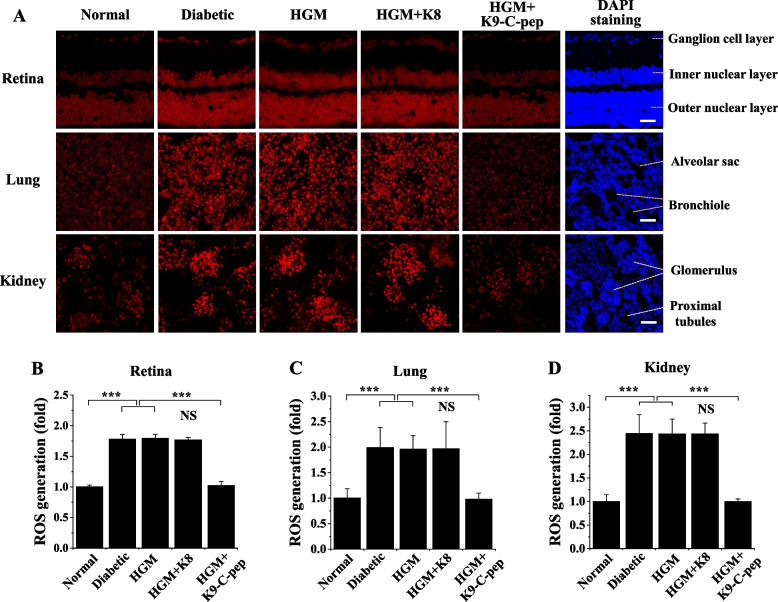
Systemic supplementation of human C-peptide via subcutaneous K9-C-peptide depots simultaneously inhibits HGM-induced ROS generation in the retinas, lungs, and kidneys of HGM mice. Six weeks after streptozotocin injection, C57BL/6 diabetic mice were treated for 4 weeks with insulin (HGM), insulin and K8 polypeptide (HGM + K8), or insulin and K9-C-peptide (HGM + K9-C-pep), as illustrated in Fig. 1A. Normal, non-supplemented diabetic, and HGM mice underwent sham operations as controls. ROS generation in the retina, lung, and kidney was visualized by confocal microscopy with dihydroethidium staining. **A** Representative fluorescence images of ROS generation. The nuclei were stained with DAPI to visualize cellular distribution in the sections. Scale bar, 50 µm. Quantification of ROS generation in the retina (**B**), lung (**C**), and kidney (**D**) sections by measuring the fluorescence intensity (*n* = 6). Statistical significance was determined using one-way ANOVA with Holm-Sidak’s multiple comparisons test. ***P < 0.001. NS, non-significant

Because persistent ROS generation and TGase activation were reported to be important in the development of HGM-induced aortic vascular dysfunction [17], we studied persistent ROS generation and TGase activation in the retina, lung, and kidney of HGM mice and the inhibitory effects of K9-C-peptide on these pathophysiological events. Hyperglycemia stimulated ROS generation in the retinas of diabetic mice, and this stimulation persisted after blood glucose normalization (Fig. 2A, B). The persistent ROS generation was inhibited by K9-C-peptide, but not by K8 polypeptide (Fig. 2A, B). Hyperglycemia also induced persistent ROS generation in the lungs and kidneys of HGM mice, which was normalized by K9-C-peptide (Fig. 2A, C, D).

**Fig. 3:**
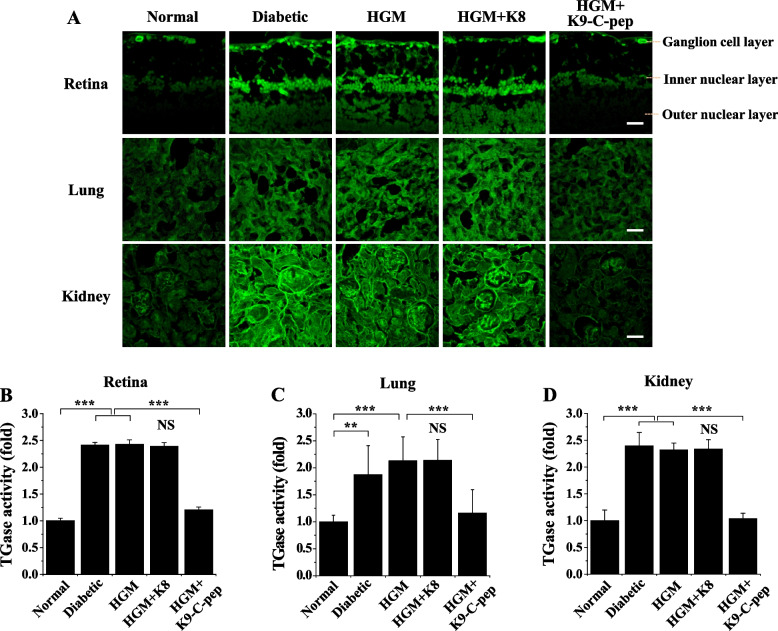
Systemic human C-peptide supplementation simultaneously inhibits HGM-induced TGase activation in the retina, lung, and kidney of HGM mice. C57BL/6 diabetic mice were treated for 4 weeks with insulin (HGM), insulin and K8 polypeptide (HGM + K8), or insulin and K9-C-peptide (HGM + K9-C-pep). Normal, non-supplemented diabetic, and HGM mice underwent sham operations as controls. In vivo TGase activity was visualized in the retina, lung, and kidney by confocal microscopy. **A** Representative fluorescence images of TGase activity. Scale bar, 50 µm. Quantification of TGase activity in the retina (**B**), lung (**C**), and kidney (**D**) sections based on fluorescence intensity (*n* = 6). Statistical significance was determined by one-way ANOVA with Holm-Sidak’s multiple comparisons test. **P < 0.01, ***P < 0.001. NS, non-significant

We next examined the effect of C-peptide on HGM-induced TGase activity in the retina, lung, and kidney. Compared with non-diabetic mice, diabetic mice displayed elevated TGase activity, which was sustained after the mice returned to normoglycemia (Fig. 3A–D). The sustained elevation of TGase activity was alleviated by K9-C-peptide, but not by K8 polypeptide. Taken together, our results suggest hyperglycemia induces persistent ROS generation and TGase activation in the retina, lung, and kidney of HGM mice after glucose normalization, and this persistent hyperglycemic stress was inhibited by systemic C-peptide supplementation via K9-C-peptide depots.

### C-peptide inhibits the HGM-induced vicious cycle of ROS generation and TGase activation and subsequent endothelial permeability in HRECs

**Fig. 4:**
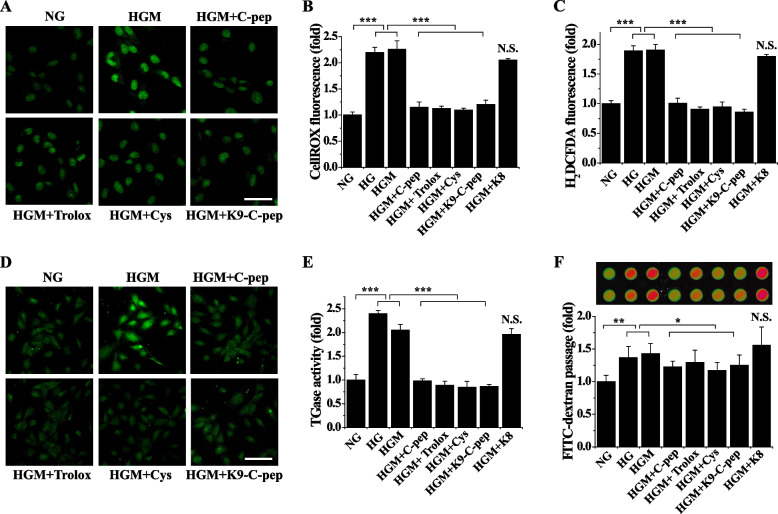
K9-C-peptide inhibits the vicious cycle of ROS generation and TGase activation and in vitro endothelial permeability in HGM-treated endothelial cells. Human retinal endothelial cells were incubated with normal glucose (NG) or high glucose (HG) for 6 days or high glucose for 3 days followed by normal glucose for 3 days (HGM) in the presence of 1 nM K9-C-peptide or K8 polypeptide, 0.5 µM Trolox, 50 µM cystamine (Cys), or 1 nM human C-peptide (C-pep). ROS generation and in situ TGase activity were determined by confocal microscopy. **A**–**C** Intracellular ROS levels were visualized using CellROX™ green (**A**, **B**) and H_2_DCFDA (**C**) and were quantitated by fluorescence intensity (*n* = 4). **A** Representative images of CellROX™ green. Scale bar, 50 µm. **B**,** C** Effects of inhibitors on HGM-induced ROS generation. **D** Representative images of in situ TGase activity. Scale bar, 100 µm. **E** Effects of inhibitors on HGM-induced TGase activation (*n* = 4). **F** A representative fluorescence image of in vitro endothelial cell monolayer permeability. In vitro endothelial permeability was quantitated by measuring FITC-dextran passage (*n* = 4). Statistical significance was determined by one-way ANOVA with Holm-Sidak’s multiple comparisons test. *P < 0.05, **P < 0.01, ***P < 0.001. NS, non-significant

To study the role of persistent hyperglycemic stress in vascular dysfunction, we tested whether C-peptide can inhibit HGM-induced vascular permeability in HRECs by arresting persistent ROS generation and TGase activation. Exposure of endothelial cells to high glucose conditions elevated intracellular ROS levels and TGase activities, which persisted after glucose normalization (Fig. 4A–E). Consistent with our in vivo results, the persistent ROS generation and TGase activation were inhibited by human C-peptide, as well as by both the ROS scavenger Trolox and the TGase inhibitor cystamine, demonstrating they were in a vicious cycle (Fig. 4A–E). Trolox is a water-soluble analog of vitamin E, while cystamine is an organic disulfide. Moreover, this persistent hyperglycemic stress was also suppressed by K9-C-peptide, but not by K8 polypeptide, suggesting the C-peptide portion of K9-C-peptide has inhibitory effects equivalent to human C-peptide on the HGM-induced vicious cycle between ROS generation and TGase activation.

We next examined the role of the ROS–TGase vicious cycle in HGM-induced endothelial permeability in HRECs. Hyperglycemia induced endothelial permeability in vitro, which persisted after glucose normalization. This HGM-increased endothelial permeability was inhibited by Trolox and cystamine (Fig. 4F). C-peptide and K9-C-peptide also inhibited the HGM-induced endothelial permeability. These results show C-peptide attenuates the HGM-induced vicious cycle between ROS generation and TGase activation, which in turn inhibits endothelial permeability in HRECs.

### C-peptide inhibits HGM-induced vascular leakage and neurodegeneration in the retinas of HGM mice

**Fig. 5:**
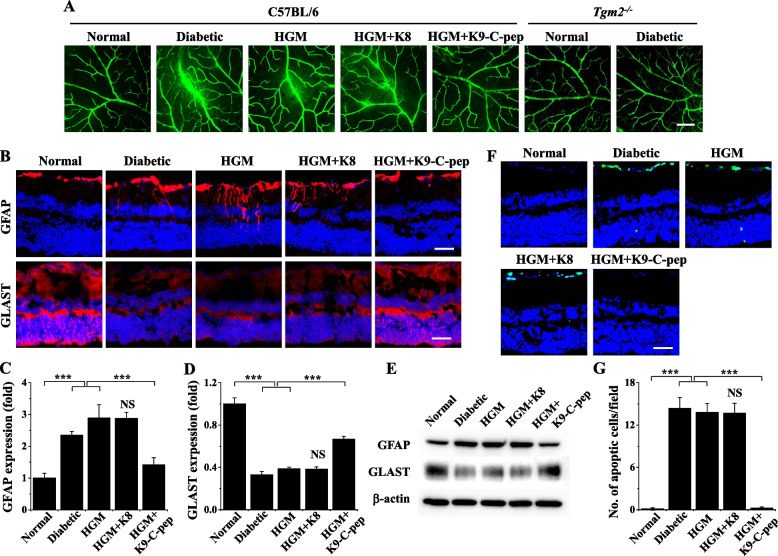
Systemic human C-peptide supplementation inhibits HGM-induced vascular leakage and neurodegeneration in the retinas of HGM mice. After insulin-supplemented diabetic mice (HGM) were subcutaneously implanted twice with K8 polypeptide (HGM + K8) or K9-C-peptide (HGM + K9-C-peptide) depots twice for total 4 weeks, vascular leakage, expression of glial fibrillary acidic protein (GFAP) and glutamate aspartate transporter (GLAST), and neuro-apoptosis were analyzed by immunofluorescence or Western blotting in mouse retinas. **A** Visualization of vascular leakage by fluorescence angiography using FITC-dextran in the whole-mounted retinas of C57BL/6 and TGase2-null (*Tgm2*.^*−/−*^) mice. Scale bar, 50 µm.** B** Visualization of GFAP and GLAST expression (red) with nuclear counterstaining using DAPI (blue) in retinal sections. Scale bar, 50 µm. Quantification of GFAP (**C**) and GLAST (**D**) expression based on fluorescence intensity (*n* = 6). **E** GFAP and GLAST expression was analyzed by Western blotting. **F** Visualization of TUNEL-positive cells (green) with nuclear counterstaining (blue). Scale bar, 50 µm. **G** Quantification of apoptotic cells (*n* = 6). Statistical significance was determined by one-way ANOVA with Holm-Sidak’s multiple comparisons test. NS, non-significant. ***P < 0.001

Because C-peptide inhibited HGM-induced endothelial permeability in HRECs, we supplemented HGM mice systemically with C-peptide using K9-C-peptide depots and explored the inhibitory effects on HGM-induced retinal, pulmonary, and renal dysfunctions. We first investigated the effect of C-peptide on retinal microvascular leakage and neurodegeneration in HGM mice. Diabetic mice displayed high levels of extravasation of FITC-dextran in the retina compared with normal mice, and this retinal microvascular leakage persisted after glucose normalization in HGM mice (Fig. 5A). Furthermore, the persistent microvascular leakage was inhibited by K9-C-peptide, but not by K8, in the retinas of HGM mice (Fig. 5A). Microvascular leakage was not detectable in the retinas of diabetic TGase2-null (*TGM2*^−/−^) mice compared with those of non-diabetic *TGM2*^−/−^ mice, implicating the role of TGase2 in retinal microvascular dysfunction (Fig. 5A).

We next examined the inhibitory effect of C-peptide on retinal neurodegeneration by analyzing apoptosis and GFAP and GLAST expression in HGM mice. In the immunofluorescence assays, compared with non-diabetic retinas, diabetic retinas displayed hyperglycemia-activated GFAP expression and extension to the outer plexiform layer, which represents activation of Müller glia and is a key pathological event of early DR (Fig. 5B, C). This hyperglycemia-induced GFAP expression persisted after glucose normalization, but it was inhibited in HGM mice by K9-C-peptide. GLAST expression was downregulated after normoglycemia in HGM mice, and this persistent downregulation was reversed by K9-C-peptide (Fig. 5B, D). Furthermore, Western blot analysis showed inhibitory effects of K9-C-peptide against HGM-induced GFAP expression and GLAST downregulation (Fig. 5E). Apoptosis, as indicated by the number of TUNEL-positive cells, was increased in diabetic retinas compared with control retinas, and the HGM-induced apoptosis was normalized by K9-C-peptide (Fig. 5F, G). K8 polypeptide had no effect on HGM-induced apoptosis or expression of GFAP and GLAST. These results show that C-peptide supplementation attenuates retinal dysfunction by suppressing persistent vascular leakage and neurodegeneration in HGM mice.

### C-peptide inhibits HGM-induced vascular leakage and fibrosis in the lungs of HGM mice

**Fig. 6:**
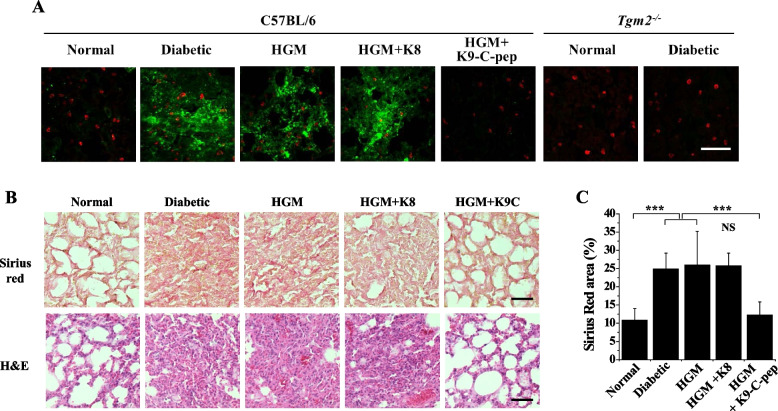
K9-C-peptide supplementation inhibits HGM-induced pulmonary vascular leakage and fibrosis in the lungs of HGM mice. HGM mice were implanted for 4 weeks with K9-C-peptide (HGM + K9-C-pep) or K8 polypeptide (HGM + K8) depots and then subjected to analysis of pulmonary vascular leakage and fibrosis. Pulmonary fibrosis was assessed by Sirius red and H&E staining. **A** Visualization of microvascular leakage using FITC-dextran in the mouse lungs of C57BL/6 and *Tgm2*.^*−/−*^ mice. Scale bar, 100 µm. **B** Representative images of lung tissues stained with Sirius red and H&E. Scale bar, 100 µm. **C** The positive area of Sirius red staining (*n* = 6). Statistical significance was determined by one-way ANOVA with Holm-Sidak’s multiple comparisons test. NS, non-significant. ***P < 0.001

Because microvascular leakage was reported to lead to pulmonary fibrosis [8], we investigated the inhibitory effects of C-peptide on HGM-induced microvascular leakage and fibrosis in the lungs of HGM mice. Hyperglycemia induced vascular leakage in the lungs of diabetic mice, which persisted after blood glucose normalization in HGM mice (Fig. 6A). This persistent vascular leakage was inhibited by K9-C-peptide, but not by K8 polypeptide. In contrast, hyperglycemia did not induce pulmonary vascular leakage in diabetic *Tgm2*^−/−^ mice compared with non-diabetic *Tgm2*^−/−^ mice. Furthermore, histopathological analysis of H&E-stained pulmonary tissues revealed hyperglycemia-induced pulmonary fibrosis in the lungs of diabetic mice, which was sustained after the restoration of normoglycemia in HGM mice (Fig. 6B, C). This HGM-induced pulmonary fibrosis was alleviated by K9-C-peptide, but not by K8 polypeptide. These results suggest that C-peptide inhibits pulmonary dysfunction by inhibiting microvascular leakage and fibrosis in HGM mice.

### C-peptide inhibits HGM-induced glomerular VE-cadherin disruption and vascular leakage in HGM mice

**Fig. 7:**
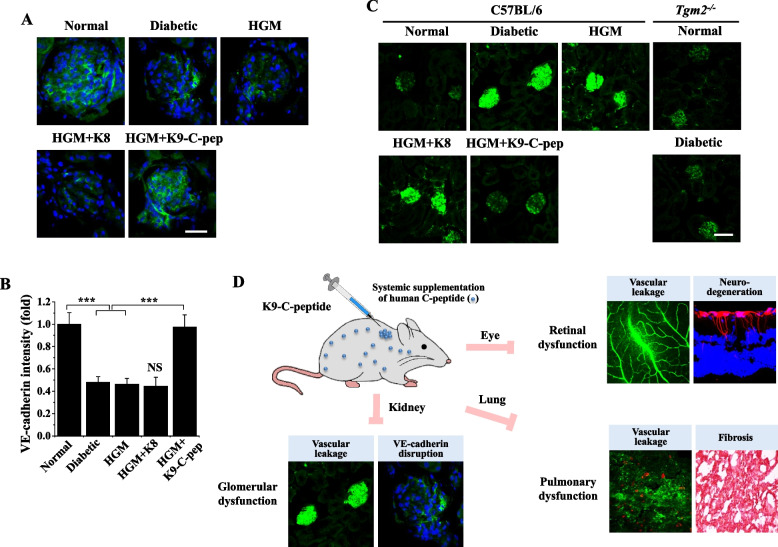
Systemic human C-peptide supplementation inhibits HGM-induced glomerular VE-cadherin disassembly and vascular leakage in the kidney of HGM mice and a schematic diagram depicting simultaneous inhibitory effects of K9-C-peptide against HGM-induced retinal, pulmonary, and glomerular dysfunction. HGM mice were implanted for 4 weeks with K9-C-peptide (HGM + K9-C-pep) or K8 polypeptide (HGM + K8) depots and then subjected to analysis of glomerular VE-cadherin disassembly and vascular leakage in the kidney. **A** Visualization of VE-cadherin with nuclear counterstaining using DAPI (blue) in the kidney. Scale bar, 50 µm. **B** Adherens junctions were quantified by based on fluorescence intensities of VE-cadherin in glomeruli (*n* = 6). **C** Visualization of glomerular vascular leakage in the kidney of C57BL/6 and *Tgm2*^*−/−*^ mice. Scale bar, 25 µm. Statistical significance was determined by one-way ANOVA with Holm-Sidak’s multiple comparisons test. NS, non-significant. ***P < 0.001. **D** Schematic model depicting simultaneous inhibitory effects of K9-C-peptide against HGM-induced retinal, pulmonary, and glomerular dysfunction in HGM mice

We further explored whether C-peptide supplementation can attenuate HGM-induced glomerular dysfunction by inhibiting VE-cadherin disruption and microvascular leakage in HGM mice. Compared with normal mice, diabetic mice displayed reduced fluorescence of VE-cadherin in the glomerulus, which represents adherens junction disassembly, and the VE-cadherin disruption was sustained in the glomerulus after blood glucose normalization (Fig. 7A, B). This sustained disruption of VE-cadherin was normalized by K9-C-peptide, but not by K8 polypeptide. We next studied the inhibitory effect of C-peptide on HGM-induced microvascular leakage in the glomerulus of HGM mice. Diabetic mice displayed high levels of glomerular FITC-dextran extravasation compared with normal mice; this vascular leakage was not affected by glucose normalization, although it was inhibited by K9-C-peptide, but not by K8 polypeptide (Fig. 7C). In contrast, hyperglycemia did not induce glomerular vascular leakage in diabetic *Tgm2*^−/−^ mice compared with non-diabetic *Tgm2*^−/−^ mice. These results suggest C-peptide inhibits glomerular dysfunction by inhibiting adherens junction disassembly and microvascular leakage in HGM mice. Collectively, our results reveal systemic C-peptide supplementation simultaneously attenuates retinal, pulmonary, and glomerular dysfunctions by inhibiting persistent ROS generation and TGase activation in HGM mice (Fig. 7D).

## Discussion

In the current study, we found C-peptide exerts simultaneous protective effects against retinal, pulmonary, and glomerular dysfunctions in HGM mice. HGM induced persistent ROS generation and TGase activation after normoglycemia in the retinas, lungs, and kidneys of HGM mice, and these pathophysiological events were inhibited by systemic C-peptide supplementation via subcutaneous of K9-C-peptide depots. We also demonstrated C-peptide supplementation attenuates vascular leakage in the three organs, retinal neurodegeneration, and pulmonary fibrosis. Based on these findings, we propose that systemic C-peptide supplementation using K9-C-peptide is a potential therapeutic approach to simultaneously alleviate multiple diabetic microvascular complications.

HGM, or persistent hyperglycemic stress after glucose normalization, is a pivotal phenomenon in the development of diabetic vascular complications [17, 19]. It was previously reported that a vicious cycle of persistent ROS generation and TGase2 activation plays a pivotal role in the HGM-induced expression of inflammatory adhesion molecules and apoptosis in the aorta of insulin-supplemented diabetic mice [17]. We found C-peptide inhibits the ROS–TGase vicious cycle and resultant endothelial permeability in HRECs under HGM conditions. Furthermore, C-peptide supplementation inhibited persistent ROS generation and TGase activation and ameliorated neuronal and vascular dysfunctions in the retinas, lungs, and glomeruli of HGM mice. Vascular leakage was not detectable in the retinas, lungs, and kidneys of diabetic *Tgm2*^−*/*−^ mice compared with those of non-diabetic *Tgm2*^−*/*−^ mice, implicating the role of TGase2 in microvascular dysfunction. Thus, it is likely the vicious cycle between ROS generation and TGase activation plays an important role in the pathogenesis of retinal, pulmonary, and glomerular dysfunctions in HGM mice.

Experimental and clinical studies have demonstrated blood glucose normalization does not prevent diabetic complications caused by HGM [2, 3, 17, 21]. Initial intensive glycemic control can reduce the incidence of microvascular complications [30]. For example, the UK Prospective Diabetes Study reported that intensive glucose treatment results in a persistent reduction of microvascular complications and long-term reduction of myocardial infarction risk and all-cause mortality in patients with type 2 diabetes [3, 31]. Despite these findings, Engerman and Kern showed DR is not improved by good glycemic control in diabetic dogs [32]. Furthermore, HGM was shown to play a role in the development and progression of DKD in patients with type 2 diabetes [33, 34]. The Diabetes Control and Complications Trial and the follow-up Epidemiology of Diabetes Control and Complications studies demonstrated that episodes of poor glycemic control contribute to the development of diabetic microvascular complications, including retinopathy, neuropathy, and nephropathy, in patients with type 1 diabetes even many years after glycemic control is achieved [3, 21, 22]. Moreover, high glucose-induced vascular endothelial cell apoptosis persisted after blood-glucose normalization through the vicious cycle of ROS generation and transglutaminase 2 in the aorta of diabetic mice [17, 20]. However, the role of HGM in the development of pulmonary vascular dysfunction remains unknown. We showed that HGM induces sustained pulmonary vascular leakage and fibrosis after normoglycemia is achieved, and this pulmonary dysfunction was attenuated by C-peptide supplementation in HGM mice**.** Thus, HGM may have a pivotal function in the development and progression of diabetic microvascular complications, including diabetic pulmonopathy.

The coincidence of diabetic complications, including DR, DPN, and DKD, has been reported in a number of clinical studies [9, 11, 14, 35]. Among the potential microvascular complications of diabetes, the coincidence of DR and DKD was reported to be high in epidemiological studies of both type 1 and type 2 diabetes [11, 12, 14]. Co-occurrence of microvascular complications has also been observed in diabetic animal models including diabetic mice and rats [10, 36]. Moreover, increased mortality was reported in patients with both DR and systemic vascular diseases, including DKD [13]. Therefore, it is important to target multiple diabetic complications to prevent the development of coincident microvascular complications and reduce the mortality rate among diabetic patients. However, studies of simultaneous treatment of multiple diabetic complications are limited. One recent study that evaluated the simultaneous therapeutic efficacy of minocycline, a tetracycline antibiotic with anti-inflammatory and anti-apoptotic properties, against DR, DPN, and DKD in type 1 and type 2 diabetic mice found that the drug partially ameliorated DR and DKD in type 1and type 2 diabetic mice, respectively, and failed to improve DPN in either animal model [10]. In the current study, we tried to simultaneously mitigate multiple diabetic complications in the retina, lung, and kidney of HGM mice by systemic C-peptide supplementation. Our results demonstrated that C-peptide simultaneously ameliorates HGM-induced retinal neurodegeneration and vascular leakage, pulmonary vascular leakage and fibrosis, and glomerular adherens junction disruption and vascular leakage in HGM mice. These results suggest that C-peptide supplementation might be an effective way to target multiple diabetic microvascular complications.

A number of previous studies demonstrated that C-peptide has beneficial effects against diabetic complications including DR, DKD, DPN, impaired wound healing, and cardiovascular dysfunction in diabetic animal models and patients with type 1 DM [17, 24, 25, 37]. However, C-peptide is limited in clinical applications by its short circulating half-life (approximately 30 min) and costly chemical synthesis [26]. Recently, we prepared K9-C-peptide, a recombinant human C-peptide conjugated to ELP, to facilitate long-term systemic delivery of C-peptide [27]. ELPs, derived from human elastin, are a class of biodegradable biopolymers suitable for the controlled release of peptide drugs [38]. Subcutaneously injected K9-C-peptide forms a depot via thermal phase transition at body temperature, which then slowly releases human C-peptide into circulation for more than 2 weeks [26]. In the current study, we found that treatment with K9-C-peptide inhibits persistent hyperglycemic stress, including ROS generation and TGase activation, and attenuates retinal, pulmonary, and glomerular dysfunctions in HGM mice. These findings suggest that it might be possible to use K9-C-peptide for the long-term treatment of multiple diabetic complications.

## Conclusions

In conclusion, we found that hyperglycemia induced persistent ROS generation and TGase activation after blood glucose normalization in the retina, lung, and kidney of HGM mice, and these pathological events were inhibited by systemic C-peptide supplementation via subcutaneous K9-C-peptide depots. Moreover, C-peptide attenuated retinal, pulmonary, and glomerular dysfunctions in HGM mice. Our results suggest that C-peptide supplementation via subcutaneous K9-C-peptide depots is a potential therapeutic approach to target multiple diabetic complications.

## Supplementary Information

**Additional file 1.** Full length images for Western blot.

## Data Availability

The datasets generated during the current study are available from the corresponding author upon reasonable request.

## Abbreviations

DKD: Diabetic kidney disease
DM: Diabetes mellitus
DPN: Diabetic peripheral neuropathy
DR: Diabetic retinopathy
ELP: Elastin-like polypeptide
GFAP: Glial fibrillary acidic protein
GLAST: Glutamate aspartate transporter 1
H&E: Hematoxylin and eosin
HGM: Hyperglycemic memory
HRECs: Human retinal endothelial cells
OCT: Optimal cutting temperature
ROS: Reactive oxygen species
TGase2: Transglutaminase 2
