# Supplementary figures and images for: Simultaneous attenuation of hyperglycemic memory-induced retinal, pulmonary, and glomerular dysfunctions by proinsulin C-peptide in diabetes
Source: BMC Med. 2023 Feb 13;21:49. doi: 10.1186/s12916-023-02760-7 (PMC9926630; doi:10.1186/s12916-023-02760-7)

Full length images for figure 5E

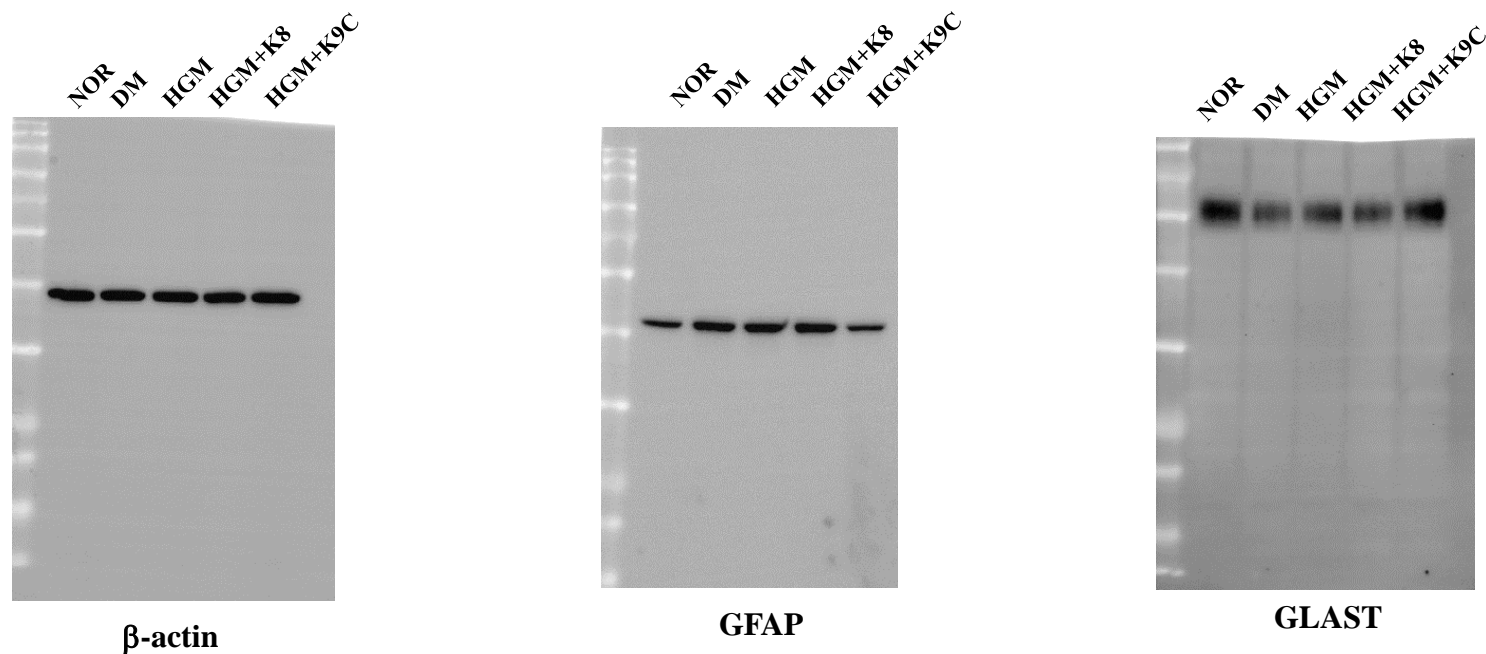

Supplement: Supplementary file 1 — Additional file 1. Full length images for Western blot. [file 12916_2023_2760_MOESM1_ESM.pdf]
